# Use of biologically-based complementary medicine in breast and gynecological cancer patients during systemic therapy

**DOI:** 10.1186/s12906-018-2325-3

**Published:** 2018-09-24

**Authors:** Loisa Drozdoff, Evelyn Klein, Marion Kiechle, Daniela Paepke

**Affiliations:** Department of Gynecology and Obstetrics, University Hospital rechts der Isar, Technical University Munich, Ismaninger Str. 22, 81675 Munich, Germany

## Abstract

**Background:**

Biologically-based complementary medicines (BB-CAM) including herbs and nutritional supplements are frequently taken by breast- and gynecological cancer patients undergoing systemic therapy. The aim of this study was to analyze the use of these natural CAM methods under systemic therapy.

**Methods:**

From September 2014 to December 2014 and February 2017 to May 2017 all patients (*n*= 717) undergoing systemic therapy at the day care unit, Department of Gynecology and Obstetrics, Technical University Munich, Germany, with breast- and/or gynecological cancer were included in this survey.

The self-administered 8-item questionnaire was developed to obtain information on complementary medication intake during systemic therapy.

**Results:**

Among 448 respondents 74.1% reported to use complementary medication simultaneous to their systemic therapy. The most frequently applied methods during therapy were vitamins and minerals supplements (72.3%), medicinal teas (46.7%), phytotherapy (30.1%), and mistletoe (25.3%).

The analysis showed that various patients-, disease- and therapy characteristics like receiving chemotherapy (*p*= 0.002), and younger age (younger than 60 years; *p*=0.017) are significantly associated with BB-CAM use.

**Conclusions:**

Our data suggest that female cancer patients undergoing systemic therapy frequently use BB-CAM medicine. Therefore, it is indispensable to implement counseling and evidence-based complementary treatments into clinical routine of cancer centers. A counseling service for integrative medicine concepts and an outpatient program (ZIGG) was therefore implemented in our cancer center in 2013. Further research on the CAM intake of cancer patients is needed in order to verify drug interactions and implement specific guidelines for integrative medication concepts.

**Electronic supplementary material:**

The online version of this article (10.1186/s12906-018-2325-3) contains supplementary material, which is available to authorized users.

## Background

Herbal medicine, nutritional supplements, acupuncture and many more therapies, also known as complementary and alternative medicines (CAM), have become increasingly popular and a common self-medication tool [1–5].

Furthermore, several studies have shown a great prevalence of CAM therapies in cancer patients [1–17]. The interpretation and identification of reliable data on the prevalence of complementary therapy methods is still difficult, as a consensus on the definition and terminology of CAM is still missing. [18] Ott et al. define *conventional* treatments as accepted and practiced by the mainstream medical community, *complementary* therapies as used in addition to conventional treatments, and *alternative* treatments as being used instead of conventional treatments. The best of conventional and complementary therapies are combined in integrated treatments [19].

But not only the diversity of the terminology of CAM is problematic when interpreting current literature, also the heterogeneity of considered therapies or medications makes data analysis challenging. Complementary therapy methods include a wide range of approaches and products, with some authors including only herbal medications, while others also include dietary supplements and mind-body practices. This is one reason for the enormous variability of CAM use in literature among cancer patients. This is the reason why we chose the specialized term of BB-CAM.

The prevalence of cancer patients using CAM differs from 50 to 70% in Germany, 45–49% in Australia, and up to 95% in the USA [7, 9, 15–17]. A European survey conducted by Molassiotis A. et al., demonstrated that the use of CAM in cancer patients in the EU is approximately 36%. Interestingly, the percentage can be up to 90% in subgroups of cancer patients [11]. Looking at characteristics for CAM users, the data shows that female sex, young age, higher educational level and a non-metastatic disease is more often associated with CAM use [20, 21] In summary, terminology, definition and also the therapy phase is relevant for a systematic analysis of complementary health approaches in cancer patients.

The National Center for Complementary and Integrative Health (NCCIH) classified CAM treatments into two subgroups: natural products (biologically–based complementary and alternative medicine, BB-CAM), which includes dietary supplements, e.g. herbs, vitamins, minerals and probiotics, or mind and body practices, e.g. yoga**,** acupuncture, relaxing techniques, meditation and others. Some complementary methods such as homeopathy, Ayurveda medicine or traditional Chinese medicine do not fit into either of these two complementary health approaches [22]**.**

The aim of the present study was to systematically analyze the use of biologically-based complementary medication (BB-CAM), such as herbs, dietary supplements and homeopathy in breast and gynecological cancer patients during systemic therapy. Here, we aimed to assess detailed information about a subgroup of BB-CAM and a special patients’ cohort to increase missing evidence about this elaborate topic of CAM.

## Methods

A cross-sectional descriptive survey was used to collect data about BB-CAM treatments with a questionnaire based on the categorical classification of different BB-CAM methods. The questionnaire was designed after research of actual data on BB-CAM and in consideration of studies and publications on questionnaire design as well as on CAM especially in gynecological oncology. The result was a self-administered 8-item questionnaire. We added the survey as an Additional file 1. Initially, the questionnaire was examined by professional physicians and researchers. Afterwards, it was pre-tested in a pilot project involving 10 selected cancer patients to prove comprehensibility, in particular understanding of specific terms like globules or homoeopathic potencies. It was known that some of the selected patients were users and some non-users of BB-CAM. Finally, we designed a revised 8-item questionnaire which was approved by the Ethics Committee of the Technical University of Munich (TUM) with the project number 412/14.

We calculated the number of participants that was needed to estimate the prevalence of BB-CAM with a 95% confidence interval and a confidence level of 5.0%. The sample size calculation was based on an estimated annual population of approximately 720 patients attending the chemotherapy unit within two different 3-month-periods. On the basis of these considerations and an expected 50% prevalence of BB-CAM use, we extrapolated that 245 participants are required. With an expected response rate of 60% we had to include at least 408 patients to take part in our survey. From September 2014 to December 2014, and again from February 2017 to May 2017, the questionnaire was handed out to all patients undergoing systemic therapy at the day care unit of the Department of Gynecology and Obstetrics, University Hospital rechts der Isar, Technical University of Munich (TUM), Munich, Germany. Two different survey periods were chosen in order to identify differences in the prevalence of BB-CAM during these two periods, as CAM therapies in general have become more popular. Additionally, two different time points of questioning were chosen to account for a potential increase of attendance of our ZIGG, which is rather unique for our Interdisciplinary Breast and Gynecological Cancer Center since its implementation in 2013.

Participation was voluntary and non-anonymous. Patients were eligible if they received any anticancer treatment at present, were older than 18 years, spoke German, and were physically and mentally able to complete the questionnaire.

The patients recorded conventional co-medications and complementary medications. Routinely prescribed supportive medication, such as vitamin D or calcium supplements were excluded from the analysis. Patients’ cancer diagnosis and complete medical history including former and current cancer therapy was documented by the treating physician. Patients were classified as BB-CAM users if they used at least one complementary treatment at present.

### Data analysis

Descriptive statistics such as mean, standard deviation, median, absolute and relative frequencies were used to describe the distribution of the socio demographic and illness or treatment related characteristics of patients. Hypothesis testing on differences between BB-CAM users and non-users was performed with two-sample *t* tests and chi-square tests. The relation of patients’ age and therapy characteristics to BB-CAM use was analyzed by multivariable logistic regression. Only completely filled-out questionnaires were analyzed. All analyses were conducted with IBM® SPSS® Statistics for Windows, Version 24 (IBM Corp., Armonk, N.Y., USA). Statistical analysis was done in cooperation with the Institute of Medical Informatics, Statistics and Epidemiology, TU Munich.

## Results

Four hundred forty-eight (62.5%) of 717 patients participated and completed the survey. After analyzing different time periods separately, we noticed just negligible differences and decided to collect the data and perform calculation of all patients collectively.

With respect to demographics, the cohorts mean age was 62.2 ± 12.4 years. With the exception of one man, all patients were women.

The vast majority of the 362 patients (80.8%) suffered from breast cancer as the primary cancer site.

More than one-third of the patients (171/448; 38.2%) had an early stage cancer and the majority (61.8%) a metastatic and/or recurrent disease, independent of their cancer type. Table 1 shows the distribution of the survey participants´ age and disease characteristics.

**Table 1:** Selected patients- and disease characteristics of the total study cohort with univariable analyses of BB-CAM use

CHARACTERISTICS		ALL PATIENTS		BB-CAM				*p* ^*1*^
				User		Non-user		
		Total Count	% of all patients	Count	% of Total	Count	% of Total	
		**448**	**100%**	332	74.1%	116	25.9%	
*Patients*								
Age in years (mean ± SD)		62.2 ± 12.4		61.5 ± 12.5		64.1 ± 12.1		0.378
	≤60 yrs.	193	43.1%	154	79.8%	39	20.2%	*0.017*
	>60 yrs.	255	56.9%	77	30.2%	178	69.8%	
*Disease*								0.168
Breast-Ca	Early Stage	147	32.8%	114	77.6%	33	22.4%	
	Advanced	201	44.9%	141	70.1%	60	29.9%	
	Recurrence	13	2.9%	9	69.2%	4	30.8%	
Ovarian-Ca	FIGO I-III	23	5.1%	21	91.3%	2	8.7%	
	FIGO IV	15	3.3%	9	60.0%	6	40.0%	
	Recurrence	37	8.3%	30	81.1%	7	18.9%	
Other Gyn-Ca		12	2.7%	8	66.7%	4	33.3%	
Disease state	Early Stage	171	38.2%	135	78.9%	36	21.1%	0.066
	Advanced+ Recurrence	277	61.8%	197	71.1%	80	28.9%	

^1^*p* values are not adjusted for multiplicity and have to be interpreted to be exploratory

62.1% (*n* = 278) of responding patients received CTX, whereas 43.8% (*n* = 196) of the patients were treated with antibodies, 33.5% (*n* = 150) hormone therapy, and 28.8% (*n* = 129) received treatment with bisphosphonates. Various combinations of more than one systemic therapy per patient were possible. The medians of co-medication of users and non-users were nearly similar (2.55 (non-user) vs. 2.51 (user)). The therapy related characteristics of the patients are presented in Table 2.

**Table 2:** Selected therapy characteristics of the total study cohort with univariable analyses of BB-CAM use

CHARACTERISTICS		ALL PATIENTS		BB-CAM				*p* ^*1*^
				User		Non-user		
		Total Count	% of all patients	Count	% of Total	Count	% of Total	
		448	100%	332	74.1%	116	25.9%	
Therapy line								0.053
Breast-Ca	Neoadjuvant	70	15.6%	58	82.9%	12	17.1%	
	Adjuvant	89	19.9%	64	71.9%	25	28.1%	
	Metastasis 1^st^ -line	78	17.4%	45	57.7%	33	42.3%	
	Metastasis 2^nd^ -line	50	11.2%	37	74.0%	13	26.0%	
	Metastasis 3^rd^ -line	20	4.5%	17	85.0%	3	15.0%	
	Metastasis ≥4^th^ -line	53	11.8%	42	79.2%	11	20.8%	
	Recurrence 1^st^ -line	8	1.8%	6	75.0%	2	25.0%	
	Recurrence ≥ 2^nd^ -line	5	1.1%	3	60.0%	2	40.0%	
Ovarian-Ca	Neoadjuvant	1	0.2%	1	100.0%	0	0.0%	
	Adjuvant	37	8.3%	29	78.4%	8	21.6%	
	Recurrent	37	8.3%	30	81.1%	7	18.9%	
*Therapy*								
Immune modulation		2	0.4%	2	100.0%	0	0.0%	0.402
Chemotherapy		278	62.1%	220	79.1%	58	20.9%	*0.002*
Antibodies		196	43.8%	143	73.0%	53	27.0%	0.625
Endocrine		150	33.5%	100	66.7%	50	33.3%	*0.011*
Bisphosphonates		129	28.8%	87	67.4%	42	32.6%	*0.041*

^1^*p* values are not adjusted for multiplicity and have to be interpreted to be exploratory

**Fig. 1:**
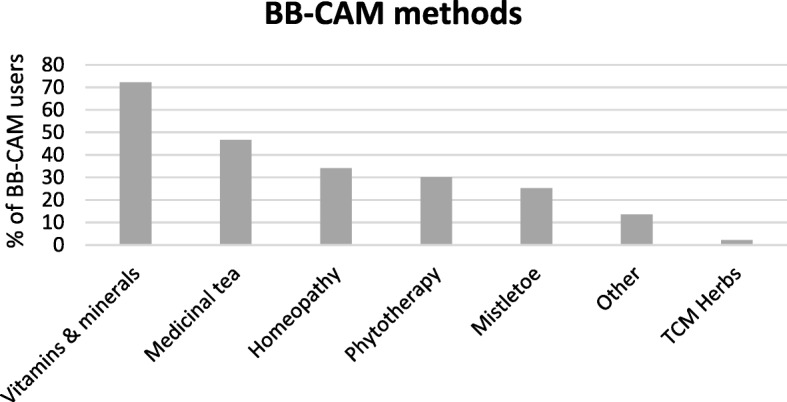
Used BB-CAM methods sorted by highest intake. Values are calculated as percentages of BB-CAM users (*n* = 332)

With respect to prevalence and predictors of BB-CAM use, the majority (74.1%; *n* = 332) of the population surveyed declared that they were currently using BB-CAM during systemic cancer therapy. As far as the different treatment types are concerned, it showed that vitamins and mineral supplements (72.3%; *n* = 240), medicinal teas (46.7%; *n* = 155), homeopathy (34.0%; *n* = 113), phytotherapy (30.1%; *n* = 100), and mistletoe (25.3%; *n* = 84) were frequently used. These findings comparing the use of different treatments are illustrated in Fig. 1.

The category “Other” (13.6%; *n* = 45) included special nutritional supplements in particular. Various combinations of more than one BB-CAM method per patient were also possible.

When evaluating the patients’ variables associated with BB-CAM use by applying univariable analysis, we noticed that 79.1% of the patients who were receiving chemotherapy used BB-CAM significantly more often (79,1 vs. 65,9%; *p* = 0,002). In this patient group especially mistletoe was used (24,8 vs. 8,8%; *p* = < 0,001). In contrast, patients receiving endocrine therapy and/or bisphosphonates as a systemic therapy applied significantly fewer complementary methods (endocrine: 66,7 vs. 77,9% p = 0,011; bisphosphonates: 67,4 vs. 76,8% *p* = 0,041). Similar results of less intake are seen when comparing the use of mistletoe treatments with endocrine or bisphosphonate medication (8.7% and 10.9%, respectively). Furthermore, significant differences become evident upon analyzing the patients´ age. Here, patients in the age group below or equal to 60 years (79.8 vs. 69.9%; *p* = 0.017) use BB-CAM significant more often than patients older than 60 years.

By analyzing patients’ age and therapy characteristics by applying multivariable logistic regression we found that patients older than 60 years had 0.61 lower odds than patients below or equal to 60 years (95% CI: 0.39–0.97; *p* = 0.038) and breast cancer patients in metastatic first-line therapy had 0.39 times lower odds than the reference (95% CI: 0.15–0.94). Logistic regression thus suggests that BB-CAM use is significantly unlikely in these patient groups (Table 3). When comparing survey years (2014 vs. 2017), only a negligible difference was seen in the prevalence of BB-CAM use (71.5 vs. 75.2%; *p* = 0.409).

**Table 3:** Bivariate logistic regression model with odds of use of BB-CAM, adjusted for selected patients´ characteristics

CHARACTERISTICS		BB-CAM USE			
		Odds ratio	95% CI		p
			Lower	Upper	
*Age (years)*					
≤60 yrs.		1.00 (Reference)			
>60 yrs.		.612	.385	.972	*.038*
*Therapy*					
Bisphosphonates	No medication	1.00 (Reference)			
	Medication	.975	.532	1.787	.934
Antibodies	No medication	1.00 (Reference)			
	Medication	.875	.503	1.521	.635
Endocrine	No medication	1.00 (Reference)			
	Medication	1.018	.547	1.895	.955
Chemotherapy	No medication	1.00 (Reference)			
	Medication	1.576	.879	2.824	.126
*Therapy line*					.390
Breast-Ca	Neoadjuvant	1.00 (Reference)			
	Adjuvant	.766	.316	1.857	
	Metastasis 1^st^ -line	**.**394	**.159**	**.973**	
	Metastasis 2^nd^ -line	.823	.302	2.242	
	Metastasis 3^rd^ -line	1.649	.384	7.073	
	Metastasis ≥4^th^ -line	.948	.355	2.530	
	Recurrence 1^st^ -line	.892	.149	5.320	
	Recurrence ≥2^nd^ -line	.372	.054	2.544	
Ovarian-Ca	Adjuvant	1.072	.371	3.099	
	Recurrent	1.035	.363	2.955	

More importantly, there were 82 patients who used BB-CAM (82/332; 24.6%) as they had been participating in a further study design. It is also noteworthy that 23.4% (*n* = 105/448) of the patients surveyed and 31.3% (*n* = 104/332) of the BB-CAM users used the opportunity of consultation for CAM usage in our clinic (ZIGG). A notable increase was seen in the attendance of ZIGG within the survey time. In 2014, 25 patients (18.2%) took part in the integrative medicine counseling service of our clinic. After 3 years, i.e. in 2017, this number increased to 80 patients (25.7%). Despite the increased ZIGG attendance (7.5%) of our patients during the two survey periods, the prevalence of BB-CAM usage in the two surveyed cohorts had not changed remarkable.

## Discussion

In addition to the growing evidence for the widespread use of CAM, our data suggest a frequent use of biologically based complementary medicine during systemic therapy among patients with breast and gynecological cancer. In our survey population three-quarters (74.1%) of the responding patients reported an ongoing use of BB-CAM. In view of the literature, this is an overall high number. CAM use may have increased in recent years. A study from 1994 [6] suggested that CAM use in gynecological cancer patients in the UK was around 16%, whereas another previous study from 2006 reported a prevalence of 40% among comparable cancer patients in Europe [13]. The group of Horneber et al. also found an increase in the prevalence of CAM use from an estimated 25% in the 1970s to about 50% in the year 2000 among patients with breast cancer [7]. This information is important for clinicians as it emphasizes that their patients frequently use CAM.

Many studies have tried to characterize a typical profile of a CAM user according to sociodemographic or disease-related data. Young female patients with a higher education suffering from breast cancer are often associated with a frequent use of CAM [8, 17, 20, 21, 23]. However, other studies failed to reveal any significant correlations between gender, cancer diagnosis, age and educational level [9, 12, 24]. Accordingly, our data suggest that CAM use is more popular among patients younger than 60 years.

Furthermore, we investigated that patients in the therapy setting of a first-line metastatic breast cancer therapy were undergoing less BB-CAM treatments than other patients. Nonetheless, our data also show that breast cancer patients in a neoadjuvant therapy setting (82.9% BB-CAM users), or patients in a metastasis third- line therapy (85.0% BB-CAM users), take BB-CAM more commonly than patients during other therapy lines. Divergent data exist concerning the association of therapy lines with CAM use. A study in 2015 reported that receiving adjuvant chemotherapy is associated with frequent CAM use [25], while Fremd et al. showed that patients in further therapy lines of metastatic breast cancer demonstrated increased CAM user rates [20]. Consequently, we could not support or falsify any of the published reports in total. The present study proposes that metastatic breast cancer patients are less willing to use BB-CAM while they are receiving first-line therapy (57.7% of the patients using BB-CAM) compared to patients during advanced therapy lines. This fact may support the observed correlation of Fremd et al. who reported higher rates of CAM user in further metastatic therapy lines.

The by far most commonly used CAM therapies are biologically-based complementary treatments. Wilkinson et al. reported vitamin/mineral supplement as the most frequently used therapy, followed by herbs, chiropractic and massage therapy [17]; in contrast, praying followed by BB-CAM were most often used in a German Comprehensive Cancer Centre [20]. Our findings confirm earlier studies reported in the literature documenting the regular use of BB-CAM [13, 17, 26, 27]. This frequent use is of some concern, as a number of herbs might interact with conventional drugs or produce a variable degree of toxicity. There is an urgent need to evaluate the effects of commonly used remedies and assess their toxicity profile. With our data serving as background, it is important to take into consideration that cancer patients expect the oncologist to be the medical provider of advice and treatment in the context of CAM [28]. In contrast, only 50% of practicing oncologists state to be interested in CAM and 77% rate their level of skills as insufficient [29]. According to recent data, 70% of the patients reported that their oncologist did not take time to discuss CAM treatment options [8]. The information of using BB-CAM is also really important for professionals, especially during an ongoing study design. We could not find any data about this variable in past researches. However, our data confirm that 24.7% of the BB-CAM users had been concomitantly participating in a further study design. This can lead to distortions of the study results and further mistakes in the future treatment of patients. Previous studies showed that information about CAM most frequently came from informal and uncontrolled sources like friends/family and media [30]. In our study cohort almost one third (31.6%) of the BB-CAM users took part a specific counseling program (ZIGG) and therefore were advised professionally.

The integrative consultation program was established at our University Hospital rechts der Isar in 2013 for gynecologic and obstetric patients to create a reliable therapy setting between CAM and conventional drugs. Gynecologists, oncologists and trained nurses work together in an interdisciplinary team to achieve the best comprehensive care for patients. Special skills in phytotherapy, homeopathy, anthroposophical medicine and other CAM treatments contribute to the indispensable know-how of professionals working in such an integrative center. The routine anamnesis should be completed by explicit questions about the use of CAM methods. Good communication skills and an open discussion about CAM issues are the key to protect patients from an inappropriate, unhelpful or even dangerous use of CAM.

One of the study’s limitations is that we did not collect more data on patients’ sociodemographic aspects and their motivations for using BB-CAM. Studies suggest that patients are looking for different benefits from CAM, for example, to improve the immune system, reduce side effects, and not miss an opportunity for well-being [26, 27]. It would be interesting for future research to analyze patients´ choice of CAM treatment and the contributing factors. Another limitation lies in the study cohort itself. Due to the fact that a structured integrative consultations program exists at the clinic, more patients are becoming aware of integrative therapies and are possibly more likely to use BB-CAMs. Furthermore, we cannot exclude recall bias, because BB-CAM intake was based on self-report.

## Conclusion

In comparison to other studies, usage of BB-CAM concomitant with systemic therapy in our department is considered to be common. Although there is a positive trend in using the opportunity of CAM counseling, there are still many patients using BB-CAM without any professional expertise at all. Further research on the safety and efficiency of CAM has to be established to base professional counseling on an extensive evidence of CAM. An implementation of standard operating procedures for CAM counseling in cancer centers and the adjustment of postgraduate medical education will be beneficial for patient management and likely to increase patient satisfaction.

## Additional file

Additional file 1: Questionnaire. This additional file contains the delivered survey for the patients of our chemotherapy unit. It includes an explanation of the survey for the patients, the questionnaire and an extra paper for additional information for the professional insight in the patients´ file. (PDF 500 kb)
