# Supplementary material for: Use of biologically-based complementary medicine in breast and gynecological cancer patients during systemic therapy
Source: BMC Complement Altern Med. 2018 Sep 24;18:259. doi: 10.1186/s12906-018-2325-3 (PMC6154925; doi:10.1186/s12906-018-2325-3)
Supplement: Supplementary file 1 — Questionnaire. This additional file contains the delivered survey for the patients of our chemotherapy unit. It includes an explanation of the survey for the patients, the questionnaire and an extra paper for additional information for the professional insight in the patients´ file. (PDF 500 kb) [file 12906_2018_2325_MOESM1_ESM.pdf]

Dear patient,

We are writing to you in regard to your treatment in our day care unit.
We would be grateful for your assistance!
We are analysing the use of complementary and alternative medicine and hereby investigating the possible risk of interactions between these medications and conventional treatments.
Please be so kind and respond to the questions placed below.
With your contribution you will help to support important research in the field of complementary and alternative medicine (CAM). Focus of this research is the simultaneous use and potential interactions of CAM therapies with conventional therapies. We would very much appreciate it if your participation in this survey.

There are two ways to return the fulfilled questionnaire:

1. You can give the fulfilled questionnaire to your attending physician during your consultation in the day care unit.
   or
2. You can send the fulfilled questionnaire directly to the study team in Munich, which will evaluate the data. The address is:

OÄ Dr. Daniela Paepke

Thank you for your help and motivation in this matter!

With your participation you allow employees or doctoral candidates of the Technical University of Munich (TUM), Klinikum rechts der Isar Munich to look into your patients' files to order to receive important information on therapy or disease related characteristics (e.g. diagnosis, actual therapy). The produced data will be used and analysed for the investigation of the already described research project exclusively.
All data regarding this questionnaire underlies professional discretion and are subject to legal requirements. The scientific analysis is carried out in encoded form. Your patients´ file stays with your attending physician in any case.

No.:

Which conventional medication do you take besides your present anticancer treatment?
Please write down the exact dosage per day. (Clexane 20 1-0-0; Omeprazol 20 1-0-1; etc.)
It is important to list all medicaments.

____

Please list all phytotherapeutical medicaments you take with stating the exact dosage per day/per week here.
(e.g. St. John's Wort 900 mg, Valerian, Mistletoe: e.g. Iscador M Serie 1 3/week s.c. etc.)

____

Please list all micronutrients and vitamins you take with stating the exact dosage. (e.g. Orthomol immun; Selen, Zinc, Vit D (z.B Vigantolette 1000IE); Vit. C, etc.)

____

Do you use herbs or medical mushrooms that belong to TCM?
Please list frequency and dosage of intake.

____

Do you use homeopathic medicaments as globules, drops or powder?
Please list all of these medications with stating the exact dosage and potency. (e.g. Pulsatilla D4 3x10 Globules;
Passiflora D1 1x15 drops for night; Apatit D6 2x1 knife points, ect.)

____

Do you drink medical teas? Please list kind and frequency of intake.

____

Do you drink juice of grapefruits or oranges? Please write down how much per day.

____

Do you take further substances, which are not listed specifically in this questionnaire?
Please list them here.

____

Thanks for your assistance!

Patient number:
Age:
Diagnosis:
Actual Therapy:
Therapy line:
Others:
